# Dendritic and mast cell involvement in the inflammatory response to primary malignant bone tumours

**DOI:** 10.1186/s13569-016-0053-3

**Published:** 2016-08-01

**Authors:** Y. Inagaki, E. Hookway, K. A. Williams, A. B. Hassan, U. Oppermann, Y. Tanaka, E. Soilleux, N. A. Athanasou

**Affiliations:** 1 Nuffield Department of Orthopaedics, Rheumatology and Musculoskeletal and Sciences, University of Oxford, Nuffield Orthopaedic Centre, Oxford, OX3 7HE UK; 2 Department of Orthopaedic Surgery, Nara Medical University, Kashihara, Japan

**Keywords:** Immunity, Bone sarcomas, Dendritic cells, Mast cells, Macrophages, Lymphocytes

## Abstract

**Background:**

A chronic inflammatory cell infiltrate is commonly seen in response to primary malignant tumours of bone. This is known to contain tumour-associated macrophages (TAMs) and lymphocytes; dendritic cells (DCs) and mast cells (MCs) have also been identified but whether these and other inflammatory cells are seen commonly in specific types of bone sarcoma is uncertain.

**Methods:**

In this study we determined the nature of the inflammatory cell infiltrate in 56 primary bone sarcomas. Immunohistochemistry using monoclonal antibodies was employed to assess semiquantitatively CD45+ leukocyte infiltration and the extent of the DC, MC, TAM and T and B lymphocyte infiltrate.

**Results:**

The extent of the inflammatory infiltrate in individual sarcomas was very variable. A moderate or heavy leukocyte infiltrate was more commonly seen in conventional high-grade osteosarcoma, undifferentiated pleomorphic sarcoma and giant cell tumour of bone (GCTB) than in Ewing sarcoma, chordoma and chondrosarcoma. CD14+/CD68+ TAMs and CD3+ T lymphocytes were the major components of the inflammatory cell response but (DC-SIGN/CD11c+) DCs were also commonly noted when there was a significant TAM and T lymphocyte infiltrate. MCs were identified mainly at the periphery of sarcomas, including the osteolytic tumour-bone interface.

**Discussion:**

Our findings indicate that, although variable, some malignant bone tumours (e.g. osteosarcoma, GCTB) are more commonly associated with a pronounced inflammatory cell infiltrate than others (e.g. chondrosarcoma. Ewing sarcoma); the infiltrate is composed mainly of TAMs but includes a significant DC, T lymphocyte and MC infiltrate.

**Conclusion:**

Tumours that contain a heavy inflammatory cell response, which includes DCs, TAMs and T lymphocytes, may be more amenable to immunomodulatory therapy. MCs are present mainly at the tumour edge and are likely to contribute to osteolysis and tumour invasion.

## Background

Host defence against primary bone sarcomas is evidenced by the presence of a chronic inflammatory cell infiltrate both within the tumour and in the ‘pseudocapsule’ around the tumour [1, 2]. The inflammatory cell response to malignant tumours, including sarcomas, is composed mainly of tumour-associated macrophages (TAMs) and lymphocytes, but is known to include dendritic cells (DCs) and mast cells (MCs) [2–5]. Tumour cells and stromal cells in the tumour microenvironment are known to release chemokines, cytokines and growth factors which attract inflammatory cells; these cells in turn release numerous humoral factors that are implicated in promoting tumour growth, angiogenesis and modulation of the immune response [5, 6].

The cellular components of the peri-tumoural inflammatory infiltrate reflect the nature of the innate and adaptive host immune response to a particular tumour. Specific inflammatory cell components have been shown to produce both positive and negative effects on tumour growth and spread. In some cancers, a high TAM infiltrate has been shown to correlate with poor prognosis, leading to the suggestion that these cells could represent a potential therapeutic target for cancer immunotherapy [7–9]. In contrast, lymphocytic infiltration, predominantly of T lymphocytes, correlates with improved survival in several cancers [10–12]. The role of other myeloid cells known to be present in cancer-related inflammation, such as DCs and MCs, is less well-defined. Some but by no means all studies have shown that DC infiltration of carcinomas is associated with increased survival and reduced incidence of metastasis; accordingly, immunotherapeutic strategies to treat solid tumours have been developed that exploit the specific role of DCs to coordinate the innate and adaptive immune response [13, 14]. MCs have been shown to play a role in regulating the adaptive immune response and in mediating vascular and stromal changes in tumour tissues [4, 15, 16]. MCs have also been shown to influence the extent of the DC, TAM and lymphocyte infiltrate through the release of mediators which enhance migration and proliferation of these cells [17–20].

Although immunotherapies targeting TAMs, lymphocytes and DCs have been investigated in some bone and soft tissue sarcomas [21–24], there has been little analysis of the number of these inflammatory cells in specific primary malignant bone tumours. In this study our aim has been to examine the nature and extent of myeloid and lymphoid inflammatory cells found in different types of primary malignant bone tumour. In this way we have sought to determine not only whether TAMs, lymphocytes, DCs and MCs are likely to play a role in influencing tumour growth and spread but also whether these cells are present in sufficient numbers to provide a target for immunotherapy in specific sarcomas.

## Methods

### Cases examined and specimen processing

Samples of 56 primary malignant bone tumours were obtained from archival surgical material at the Department of Histopathology, Nuffield Orthopaedic Centre, Oxford, UK. Samples of primary aggressive or malignant bone tumours included 14 cases of conventional osteosarcoma, 8 cases of Grade I chondrosarcoma, 4 cases of Grade II/III chondrosarcoma, 2 cases of dedifferentiated chondrosarcoma, 11 cases of giant cell tumour of bone (GCTB), 12 cases of Ewing sarcoma, 2 cases of chordoma and 3 cases of undifferentiated pleomorphic sarcoma of bone. None of the patients had received immunomodulatory neo-adjuvant therapies.

Several osteolytic benign bone tumours were also assessed for MC infiltration. These included 4 cases of fibrous dysplasia, 4 cases of non-ossifying fibroma, 2 cases of osteoblastoma, 2 cases of aneurysmal bone cyst and 2 cases of chondroblastoma.

Control tissues included samples of normal bone obtained from amputation specimens and, for mast cell staining, samples of bone obtained from two cases of systemic mastocytosis. All samples were fixed in 10 % neutral buffered formalin and decalcified in 5 % nitric acid prior to processing and embedding in paraffin wax. Serial sections of 5 µm were cut and mounted on charged microscope slides (Surgipath, UK) for immunohistochemical staining.

### Immunohistochemistry

Monoclonal antibodies employed in this study and their specificity are shown in Table 1. Sections were incubated at 37 °C for at least 24 h to improve tissue adhesion before immunohistochemistry. Tissue sections were dewaxed in xylene and rehydrated by successive immersion in graded alcohol and water. Endogenous peroxidase was blocked by treating the sections with 0.2 % hydrogen peroxide (BDH, UK) in 80 % alcohol for 30 min. Antigen retrieval was required for all antibodies. This involved microwave heat treatment in 500 ml 10 mM Tris/1 M EDTA (BDH, UK) buffer, pH 8.5.

**Table 1:** Monoclonal antibodies used in this study

Antigen	Antibody (clone)	Specificity	Source	Dilution	Pre-treatment
CD3	(F7.2.38)	T cells	Dako	1:25	MW 20 min
CD11c	(5D11)	DCs, histiocytes	Novocastra	1:100	MW 20 min
CD14	(7)	Macrophages, monocytes	Novocastra	1:50	MW 20 min
CD20	(L26)	B cells	Dako	1:100	─
CD45	(PD7/26;2B11)	Leucocyte common antigen	Dako	1:100	MW 10 min
CD68	(KP1)	Macrophages, monocytes, neutrophils, NK cells	Dako	1:1000	MW 10 min
DC-SIGN	(120,507)	Immature DCs	R&D Systems	1:500	MW 10 min
S100	(polyclonal)	DCs, histiocytes, chondrocytes	Dako	1:1000	─
Mast cell tryptase	AA1	Mast cells	Dako	1:100	MW 10 min

*MW* microwave, – no pre-treatment required

Immunohistochemistry was performed using an indirect immunoperoxidase technique with 3,3-diaminobenzidine chromogen (ChemMate Envision, Dako, UK). Sections were incubated with primary antibodies for 30 min at room temperature followed by 30 min incubation with labelled polymer and 10 min in 3,3-diaminobenzidene. The sections were then counterstained using Mayer’s haematoxylin (RA Lamb, UK) for 3 min and blued in 2 % hydrogen sodium carbonate (BDH, UK).

### Histological analysis

Areas of tumour containing the maximum inflammatory response were scored, as previously described, by a modified semi quantitative technique as sparse (<2 %), moderate (2–10 %) or dense (>10 %) after counting 500 cells in five high-powered (×400) fields and determining the number of cells expressing CD45 (leukocyte common antigen) [25, 26]. Areas of tumour necrosis were not included in this assessment. In parallel sections stained with immunophenotypic markers, identifying B and T lymphocytes, TAMs, MCs and DCs (Table 1), the number of these infiltrating cells was scored relative to the number of CD45+ cells as: +low (<5 % positive cells); ++moderate (6–25 % positive cells); +++heavy (>25 % positive cells).

## Results

Results of the immunohistochemical findings are summarised in Table 2.

**Table 2:** Tumour-infiltrating leucocyte antigen expression

	CD45	CD68	CD14	CD3	CD20	DC-SIGN	Mast cell^a^ tryptase
Giant cell tumour of bone	Dense	+++	++	+	–	+	+/++
Osteosarcoma; Undifferentiated pleomorphic sarcoma dedifferentiated chondrosarcoma	Moderate	++	+/++	+	–	+	+/++
Ewing sarcoma Chondrosarcoma Chordoma	Sparse	+/++	+/++	−/+	–	−/+	+/++

Inter/intra-tumour variability of leucocyte antigen expression noted. For details, see “Results” section

^a^Mast cells (+/++) were identified at the periphery of all osteolytic bone tumours

**Fig. 1:**
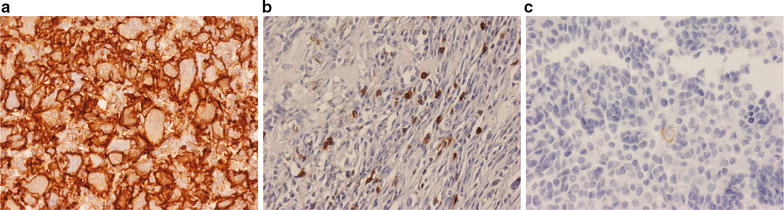
Immunohistochemistry for the leucocyte marker CD45 showing that the inflammatory cell infiltrate is: **a** heavy in GCTB. **b** Moderate in osteosarcoma. **c** Sparse in Ewing sarcoma

The extent of the CD45+ inflammatory cell infiltrate in the malignant bone tumours analysed was highly variable, not only between different types of bone sarcoma but also between sarcomas of a single type. The inflammatory infiltrate was not uniformly distributed throughout these tumours; all GCTBs contained a dense CD45+ inflammatory infiltrate while osteosarcoma and undifferentiated pleomorphic sarcomas often contained areas with a moderate CD45+ infiltrate (Fig. 1). In contrast, chondrosarcomas, chordomas and Ewing sarcomas contained a sparse inflammatory cell infiltrate. In all bone sarcomas, CD68+ TAMs comprised the largest sub-population of inflammatory cells in the tumour followed by CD3+ T cells and DCs. CD20+ B cells were absent or very rarely identified in the bone sarcomas analysed.

**Fig. 2:**
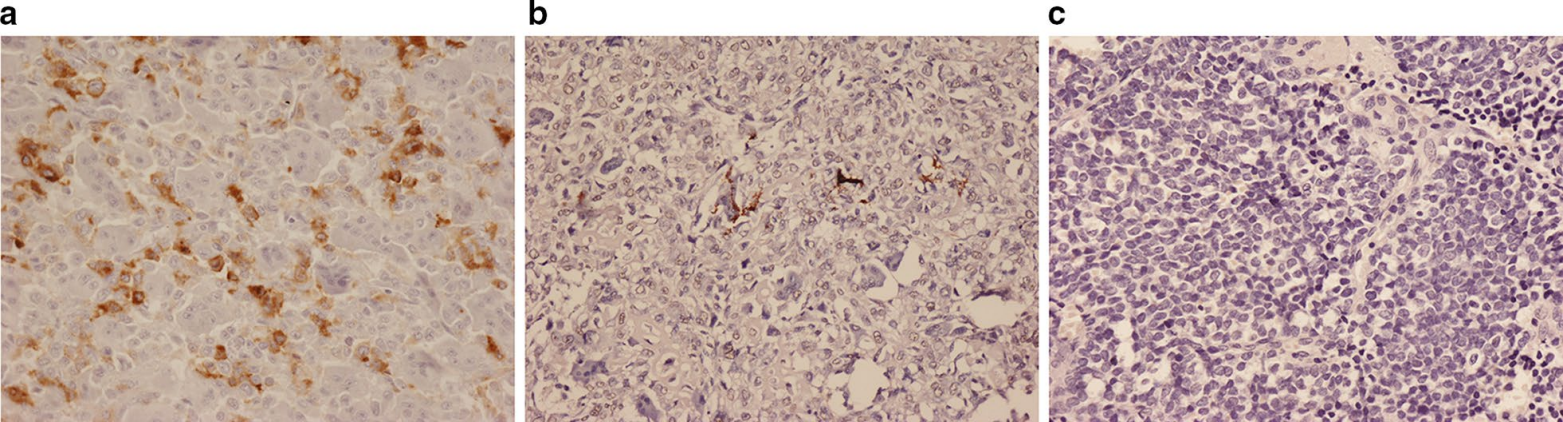
Immunohistochemistry for the DC marker for DC-SIGN showing: **a** moderate (++) expression in GCTB. **b** Low (+) expression in osteosarcoma. **c** Absence of expression in Ewing sarcoma

**Fig. 3:**
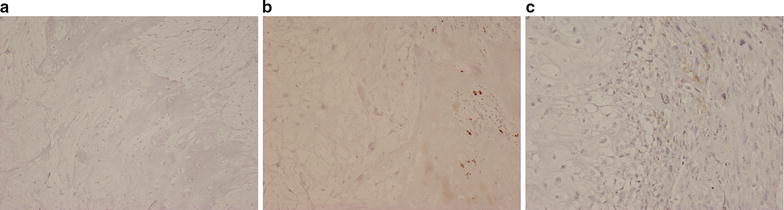
Immunohistochemistry for the macrophage marker CD68 in chondrosarcoma showing: **a** absence of TAMs in low-grade chondrosarcoma. **b** Scattered (+) TAMs in areas of matrix mineralisation within the tumour. **c** Absence of TAMs in the low-grade cartilaginous component (*left*) but scattered (+) TAMs in the high-grade sarcomatous component (*right*) of a dedifferentiated chondrosarcoma

Where there was a high number of CD68+ TAMs and CD3+ T lymphocytes in bone sarcomas, DC-SIGN-expressing DCs were noted; some of these cells stained for CD11c and S100 but they did not stain for CD1a. The most common pattern seen in conventional osteosarcoma, undifferentiated pleomorphic sarcoma and GCTB was that of a moderate or heavy (++/+++) TAM infiltrate, with a low/moderate (+/++) CD3+ T cell and DC infiltrate (Fig. 2). DCs were absent or present in low numbers (+) in chondrosarcoma, chordoma and most but not all Ewing sarcomas. All Grade 1 chondrosarcomas contained a sparse inflammatory cell infiltrate (Fig. 3a). A few (+) TAMs were noted in areas of matrix degeneration or mineralisation but there were few or no T cells or DCs identified in the tumour stroma. Grade II and III chondrosarcomas also contained few or no inflammatory cells, although some TAMs, T cells and DCs were seen in areas of matrix degeneration in fibrous tissue separating lobules of tumour cartilage. DCs were not present in the low-grade cartilaginous component but were seen in the spindle cell component of dedifferentiated chondrosarcoma (Fig. 3c). These tumours also contained numerous TAMs and T cells.

**Fig. 4:**
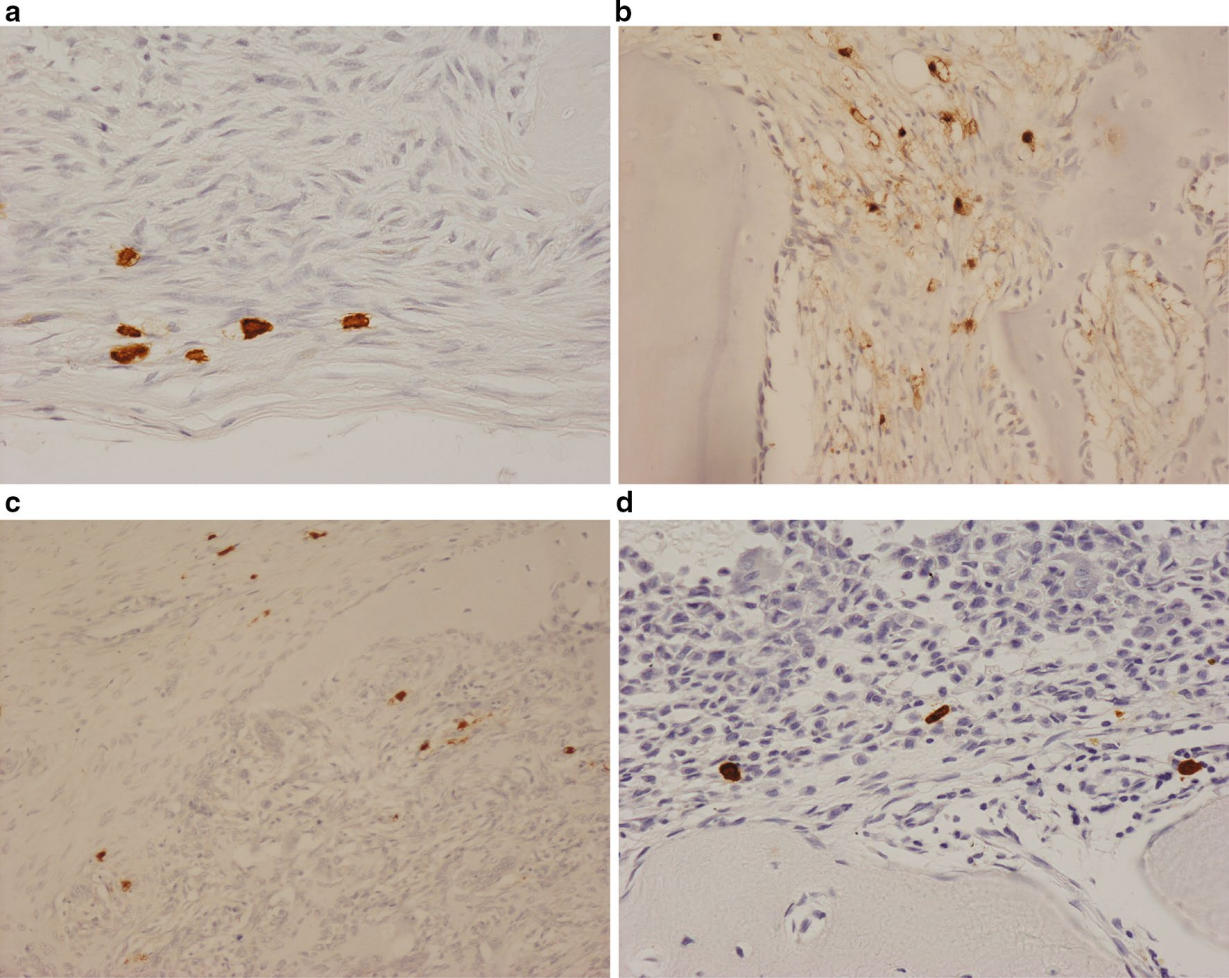
Immunohistochemistry for mast cell tryptase showing MCs in: **a** the pseudocapsule and **b** tumour-bone interface of an osteosarcoma. **c** The soft tissue margin of an aggressive GCTB. **d** The osteolytic margin of a chondroblastoma

MCs were not present or very few in number in the main tumour mass of most bone sarcomas. However, MCs (+/++) were commonly found at the periphery of invasive bone tumours in the fibrous pseudocapsule at the soft tissue margin and at the host bone-tumour interface where there was osteolysis (Fig. 4). The finding of an MC association with tumour osteolysis in sarcomas prompted us to examine MC infiltration in lytic benign bone tumours. We found that MCs were commonly sited at the host bone-tumour interface in all cases of chondroblastoma (Fig. 4d), osteoblastoma, aneurysmal bone cyst, non-ossifying fibroma and fibrous dysplasia. A few (+) MCs were also noted in fibrotic areas of undifferentiated pleomorphic sarcomas and osteosarcomas of bone. MCs (++) were also commonly seen in the fibrous component of benign bone lesions such as non-ossifying fibroma and fibrous dysplasia.

## Discussion

This study has shown that myeloid and lymphoid cells, which play a role in innate and adaptive immunity are present in the inflammatory response to primary aggressive/malignant bone tumours. A significant inflammatory cell infiltrate was commonly seen in osteosarcoma, undifferentiated pleomorphic sarcoma, GCTB and dedifferentiated chondrosarcomas. Inflammation was less pronounced in Ewing sarcoma, chordoma and chondrosarcoma. TAMs formed the major component of the inflammatory cell infiltrate in all bone tumours; there was also a variable population of CD3+ T cells and DCs. In general, the extent of DC infiltration corresponded to that of TAMs and T cells. MCs were found mainly at the osteolytic bone-tumour interface in bone sarcomas as well as other benign primary bone tumours.

As in carcinomas and soft tissue sarcomas, the major subpopulation of leukocytes noted in bone sarcomas was that of TAMs [5, 26]. TAMs have been shown to promote tumour progression in several carcinomas by suppressing the immune response, increasing expression of matrix metalloproteinases and promoting tumour angiogenesis [5, 7–9]. The role of TAMs in bone sarcomas has not been extensively investigated. TAMs have been associated with reduced metastasis and improved survival in high-grade osteosarcoma [30]. In contrast, TAM infiltration has been associated with a poor prognosis in Ewing sarcoma [31]. Osteoclasts, which carry out the bone resorption associated with growth of a bone tumour are derived from TAMs by a RANKL-dependent mechanism [32, 33], and it would be expected that a heavy TAM infiltrate would promote tumour growth and spread. Ewing sarcoma and osteosarcoma tumour cells have been shown to express RANKL and to support macrophage-osteoclast differentiation [33–36]. Although we found that the majority of Ewing sarcomas in our study induced a relatively low inflammatory cell response, we noted that most of the cells in the infiltrate were TAMs. Paradoxically, it has been shown in some reports that there is an inverse relationship between the number of osteoclasts and the extent of pulmonary metastasis [37, 38]. Further studies are required to establish the role of TAMs in high-grade bone sarcomas as these cells constitute the major subpopulation of inflammatory cells in these tumours.

DCs and CD3+ T lymphocytes were noted in all types of bone sarcoma and in general their number corresponded with the extent of TAM infiltration. DCs are efficient antigen-presenting cells that have the ability to prime naïve T cells and initiate a specific immune response [37, 38]. DCs exist in two functionally and phenotypically distinct stages, immature and mature. Immature DCs have high endocytic activity, specialise in antigen capture and processing and reside in peripheral tissues. Upon exposure to pathogen-derived products or innate pro-inflammatory signals, DCs lose their phagocytic activity and migrate to draining lymph nodes where they become mature DCs. Mature DCs have high antigen-presenting capability and interact with antigen-specific T cells to initiate a specific immune response. In tumours there is a disturbance of DC differentiation, survival and function [13, 14, 38]. A C-type lectin, a DC-specific ICAM-grabbing non-integrin (DC-SIGN), has been identified and shown to mediate strong adhesion between DCs and intracellular adhesion molecule 3 (ICAM-3) on resting T cells [39, 40]. We noted that CD3+ T lymphocytes were more commonly found in osteosarcomas than Ewing sarcomas although considerable variation was observed regarding the density and distribution of these cells, some of which have been shown to be cytotoxic against osteosarcoma tumour cells and to promote adaptive antitumor immunity in Ewing sarcoma [41, 42]. Tumour-derived factors such as vascular endothelial growth factor can induce immature DCs from the bone marrow to migrate to osteosarcomas [43, 44]. Although showing some variability, fewer DCs were seen in Ewing sarcomas where the TAM and T-lymphocyte response was also reduced. The suppression of DC antigen and function can induce immune tolerance to tumour antigens in sarcomas [39]; specifically, the alteration of carbohydrates on the cell surface is thought to influence the interaction between C-type lectins on DCs and tumour cells, thus interfering with antigen presentation [45]. DC-SIGN is a useful immunohistochemical marker of immature and mature DCs and may be useful in assessing the efficacy of DC-based immunotherapy against sarcomas [46, 47]. Antibodies targeting DC-SIGN have been used to induce a T cell response, and vaccines comprising autologous antigen-loaded DCs have been shown to prime tumour immunity [48, 49].

MCs are found throughout the body and are usually located in connective tissues, particularly around blood and lymphatic vessels as well as peripheral nerves; they are present in low numbers in normal human bone marrow [50]. MCs are mobilised to infiltrate tumours by means of stem cell factor (SCF) [16, 18]. Production of SCF and the receptor for SCF (c-kit) have been identified in several bone sarcomas, including Ewing sarcoma and osteosarcoma [51–53]. Mechanisms whereby MCs may influence tumour progression include stimulation of the release of growth factors essential for tumour growth and suppression of the host immune response to tumour cells [16, 18]. MC-derived humoral factors are known to influence the migration and function of TAMs, T-lymphocytes and DCs [18–20]. We noted that MCs were found mainly at the tumour-bone interface where there was tumour-associated osteolysis. MCs have been identified at the periphery of tumours in aggressive breast cancer and malignant melanoma [16]. MCs are known to promote osteoclastic bone resorption and to play a role in inflammatory osteolysis, renal osteodystrophy and other bone conditions where there is increased remodelling activity [50, 54]. Mediators produced by MCs which promote osteoclastic resorption include the prostaglandins E2 and D2, and cytokines such as tumour necrosis factor alpha (TNFα) [16, 19]. The latter has been shown to simulate osteoclast formation by a RANKL-independent mechanism [55], it has been shown that TAM-osteoclast differentiation in Ewing sarcoma can be induced by TNFα [33]. This may play a role in promoting tumour metastases in Ewing sarcoma which is modulated by stem cell factor and its receptor c-kit [56].

## Conclusions

Our findings show that the inflammatory response to primary malignant bone tumours is variable and to some extent depends on tumour type. TAMs and T-lymphocytes are the major inflammatory cell types seen in bone sarcomas but there is also a significant DC component within some tumours. MCs are likely to contribute to tumour osteolysis as they are almost exclusively found at the tumour-bone interface. Using specific myeloid and lymphoid inflammatory cell subpopulations as targets for immunomodulatory therapy has been proposed for bone sarcomas but the efficacy of such treatment is likely to depend of the presence of a significant inflammatory response in the tumour; our findings show that this can be highly variable, and it may thus be useful to assess the extent and nature of the inflammatory cell response in a bone sarcoma before instituting this type of therapy.

## Abbreviations

TAMs: tumour-associated macrophages
DCs: dendritic cells
MCs: mast cells
GCTB: giant cell tumour of bone
DC-SIGN: DC-specific ICAM-grabbing non-integrin
SCF: stem cell factor
TNFα: tumour necrosis factor alpha
